# Molecular Cloning, Characterization and Expression Analysis of Two Members of the Pht1 Family of Phosphate Transporters in *Glycine max*

**DOI:** 10.1371/journal.pone.0019752

**Published:** 2011-06-15

**Authors:** Zhaoyun Wu, Jinming Zhao, Ruifang Gao, Guanjun Hu, Junyi Gai, Guohua Xu, Han Xing

**Affiliations:** 1 National Center for Soybean Improvement, National Key Laboratory of Crop Genetics and Germplasm Enhancement, Nanjing Agricultural University, Nanjing, Jiangsu, China; 2 State Key Laboratory of Crop Genetics and Germplasm Enhancement, College of Resources and Environmental Sciences, Nanjing Agricultural University, Nanjing, China; University of South Florida, United States of America

## Abstract

**Background:**

Phosphorus is one of the macronutrients essential for plant growth and development. The acquisition and translocation of phosphate are pivotal processes of plant growth. In a large number of plants, phosphate uptake by roots and translocation within the plant are presumed to occur via a phosphate/proton cotransport mechanism.

**Principal Findings:**

We cloned two cDNAs from soybean (*Glycine max*), *GmPT1* and *GmPT2*, which show homology to the phosphate/proton cotransporter PHO84 from the budding yeast *Saccharomyces cerevisiae*. The amino acid sequence of the products predicted from *GmPT1* and *GmPT2* share 61% and 63% identity, respectively, with the PHO84 in amino acid sequence. The deduced structure of the encoded proteins revealed 12 membrane-spanning domains with a central hydrophilic region. The molecular mass values are ∼58.7 kDa for GmPT1 and ∼58.6 kDa for GmPT2. Transiently expressed GFP–protein fusions provide direct evidence that the two Pi transporters are located in the plasma membrane. Uptake of radioactive orthophosphate by the yeast mutant MB192 showed that GmPT1 and GmPT2 are dependent on pH and uptake is reduced by the addition of uncouplers of oxidative phosphorylation. The *K*_m_ for phosphate uptake by GmPT1 and GmPT2 is 6.65 mM and 6.63 mM, respectively. A quantitative real time RT-PCR assay indicated that these two genes are expressed in the roots and shoots of seedlings whether they are phosphate-deficient or not. Deficiency of phosphorus caused a slight change of the expression levels of *GmPT1* and *GmPT2*.

**Conclusions:**

The results of our experiments show that the two phosphate transporters have low affinity and the corresponding genes are constitutively expressed. Thereby, the two phosphate transporters can perform translocation of phosphate within the plant.

## Introduction

Phosphorus is one of the most important macronutrients required for plant growth and metabolism, and is the key component of nucleic acids, phospholipids and energy-providing ATP as well as several enzymes and coenzymes. Phosphorus is involved in energy metabolism, activation of metabolic intermediates, carbon assimilation, photosynthesis, respiration, signal transduction and enzyme regulation [1], [2], [3]. In soil, plants acquire phosphorus in the form of orthophosphate (Pi) [4], [5], [6]. Phosphate is the second most frequently limiting macronutrient for plant growth mainly because it exists in the soil in complex, insoluble, inorganic and organic forms that cannot be acquired directly by the plant [4], [7]. For this reason, the concentration of Pi in soil solution can be as high as 10 µM but is present more often at concentrations as low as 1 µM [8].

Plants respond to phosphate deficiency by increasing the rate of Pi uptake by roots [4], and upregulation of the synthesis of a carrier system is believed to contribute to the observed increase of Pi acquisition [9]. There are two Pi transport systems required by plants to facilitate absorption from diverse environments and enable subsequent transportation to all of the cells and subcellular compartments within the plant. Kinetic characterization of the Pi uptake system of whole plants [10], [11] and cultured cells [12] suggests a high-affinity transport operating in the low micromolar range and a low-affinity system operating at higher concentrations (millimolar range) [7], [13], [14], [15], [16].

Because the concentration of Pi in soil solution seldom exceeds 10 µM [8], the high-affinity transport is assumed to be the predominant system responsible for Pi uptake. Thus, a number of Pi transporters might function primarily in Pi uptake at the soil–root interface, whereas the others might participate predominantly in translocation within the plant and/or transport within certain tissues or cell types. After uptake into the roots, Pi is mainly translocated symplastically to the xylem parenchyma cells, and secretion into the xylem for long-distance translocation to the shoot is facilitated by another type of transporter-like protein [8], [17]. In plants that are not Pi-deficient, most of the Pi uptake by the roots is transported in the xylem to growing leaves. In Pi-starved plants, however, the limited supply of Pi from roots to shoots is augmented by increased mobilization of stored Pi in older leaves and retranslocation to both younger leaves and growing roots, from where Pi can again be recycled to the shoot [18]. Consequently, the uptake and allocation of Pi in plants requires multiple transport systems that must function in concert to maintain homeostasis throughout growth and development [19].

Remobilization of phosphate stored in leaves has been demonstrated and the existence of a Pi transporter that facilitates this process has been inferred. Rae *et al.* have identified several genes in a barley genomic library that appear to be members of the *Pht1* gene family. The sequence of *HORvu;Pht1;6* suggested that it is also a member of the *Pht1* gene family. The estimated *K*_m_ of HORvu;Pht1;6 is 385
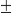
61 µM, which is characteristic of a low-affinity transporter. *HORvu;Pht1;6* is expressed in the above-ground part of the plant with strongest expression in old leaves and flag leaves and is less responsive to external concentrations of Pi, indicating that *Pht1;6* is unlikely to function in the uptake of Pi by roots from soil. Both of these organs are known to have a role in the nutrition of developing grains. The expression of *Pht1;6* in these organs suggested that it might also play a role in the remobilization of nutrients during grain development. Furthermore, *in situ* hybridization showed that *Pht1;6 is* expressed in the phloem of vascular bundles in leaves and ears. Taken together, HORvu;Pht1;6 probably functions in the remobilization of stored Pi from leaves [20]. In rice, expression of *OsPht1;2* (*OsPT2*) is increased significantly in response to Pi deficiency in root and shoot. By using transgenic rice plants expressing the GUS reporter gene, *OsPT2* was localized exclusively in the stele of primary and lateral roots. The knock-down of *OsPT2* by RNA interference significantly decreased long-distance transport of Pi from root to shoot. These data suggested OsPT2 functions in translocation of the stored Pi in the plant [21]. In conclusion, low-affinity Pi transporters have a wide range of roles in Pi uptake and translocation within the plant and are required to facilitate the movement of phosphate between subcellular compartments and organelles. However, most studies of Pi transporters in plants have focused on the roots.

Soybean (*Glycine max* L. Merr.) is one of the most economically important leguminous seed crops that provide the majority of plant proteins, and more than a quarter of the world's food and animal feed [22], [23]. To our knowledge, there is no report of soybean Pi transporters in the literature. Here, we report the characterization of two Pi transporters from soybean. The two genes are designated *GmPT1* and *GmPT2* according to the rules recommended by the Commission on Plant Gene Nomenclature. The sequences of the two genes share great similarity with that of the plant proton–Pi cotransporter. The primary functions of these genes appear to be as low-affinity Pi transporters within the plant.

## Results

### Cloning and Computational Sequence Analysis

**Figure 1:**
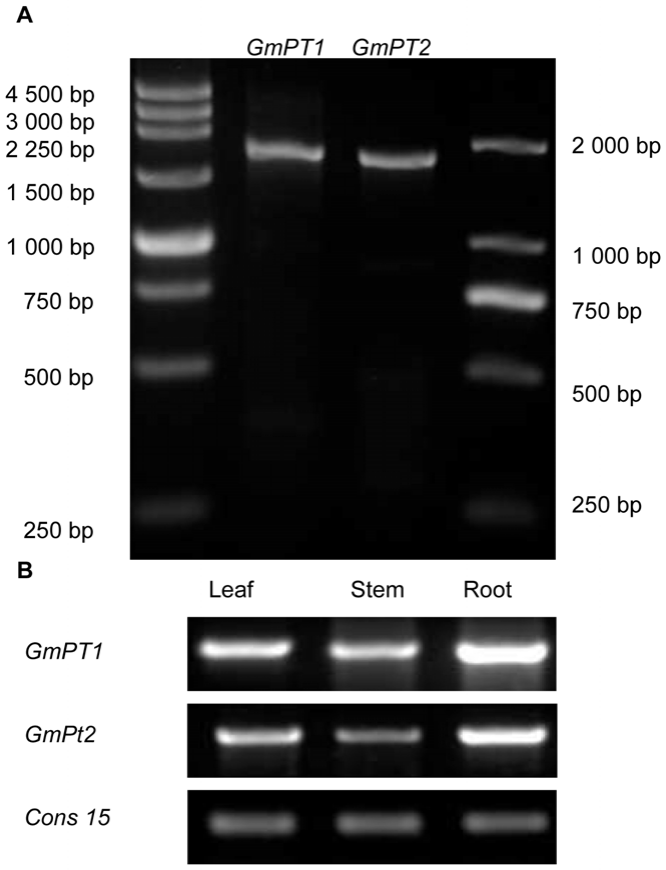
DNA gel analysis of two soybean Pi transporters. DNA gel-blot analysis of *GmPT1* and *GmPT2* (A). Lanes 2 and 3 contain *GmPT1* and *GmPT2*, respectively. The size markers are shown to the right and left of the figure. The expression profile of the two proteins in different parts of the soybean seedling (B). Seven days old soybean seedlings were used to examine the expression of *GmPT1* and *GmPT2* with *cons15* as the control.

We identified two single copy Pi transporter genes in soybean located on chromosomes Gm10 (41,391,168–41,393,008) and Gm20 (42,980,124–42,981,928). These genes are designated *GmPT1* (accession number HQ392508) and *GmPT2* (accession number HQ392509), respectively. *GmPT1* is 1841- bp long (Figure 1A) and contains an open reading frame encoding a 536 amino acid polypeptide (molecular mass 58730.46 Da). *GmPT2* is 1802 bp long (Figure 1A) and contains an open reading frame encoding a 536 amino acid polypeptide (molecular mass 58627.29 Da). Interestingly, the open reading frame in both genes spans base pairs 23–1633. These genes are 88.7% similar in nucleotide sequence and 97.9% similar in amino acid sequence. The two polypeptides share the greatest degree of similarity with the characterized Pi transporters from mouse-ear cress (*Arabidopsis thaliana*) [14], [25], tomato (*Lycopersicon esculentum* L.) [15], potato (*Solanum tuberosum* L.) [26] and barrel clover (*Medicago truncatula*) [27]. The two Pi transporters from soybean have a very high degree of identity with fungal Pi transporters from the mycorrhizal fungus *Glomus versiforme* (GvPT) and the budding yeast *Saccharomyces cerevisiae* (PHO84). GmPT1 shows 76% and 61% and GmPT2 shows 76% and 63% amino acid sequence identity with GvPT (accession number Q00908) and PHO84 (accession number P25297), respectively.

### Structure of the Soybean Pi Transporters

**Figure 2:**
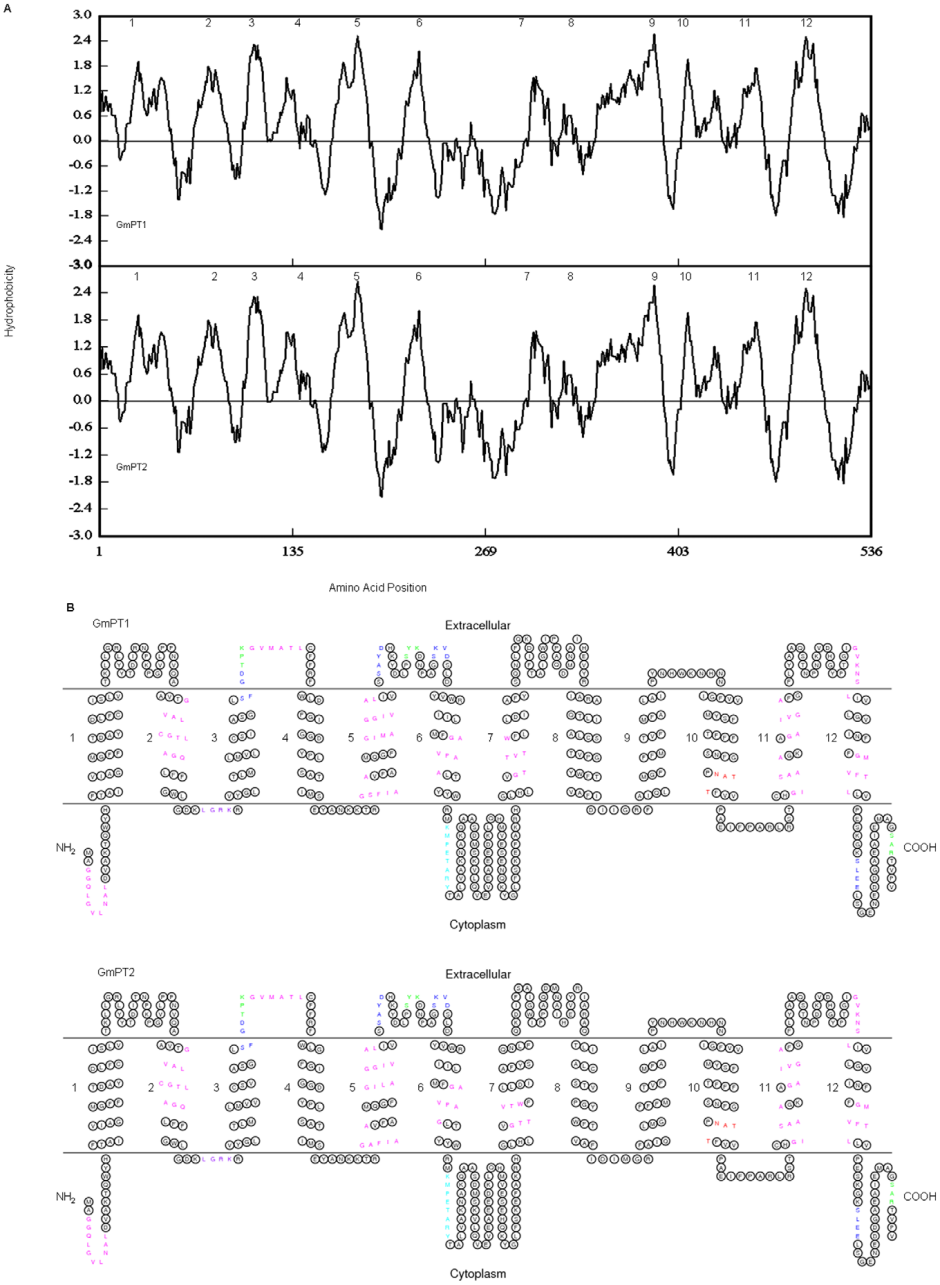
Predicted topology of GmPT1 and GmPT2. Hydrophobicity profiles of GmPT1 and GmPT2 (A). Hydropathy values for a window of 14 residues were calculated by DNAMAN version 6.0.3.93 using algorithms presented by Kyte and Doolittle [62]. Hydrophobic regions correspond to positive index numbers. The arabic numerals refer to putative membrane-spanning domains. A topological model for GmPT1 and GmPT2 (B). The membrane-spanning domains of GmPT1and GmPT2 were predicted by HMMTOP [52] and their numbering is indicated by arabic numerals 1–12. The model was drawn with the aid of TOPO2 software (http://www.sacs.ucsf.edu/TOPO2/). Enlarged symbols indicate sites of significant structure–function importance: red, N-glycosylation; green, protein kinase C phosphorylation; blue, casein kinase II phosphorylation; cyan, tyrosine kinase phosphorylation; purple, Amidation; and magenta, N-myristoylation.

**Figure 3:**
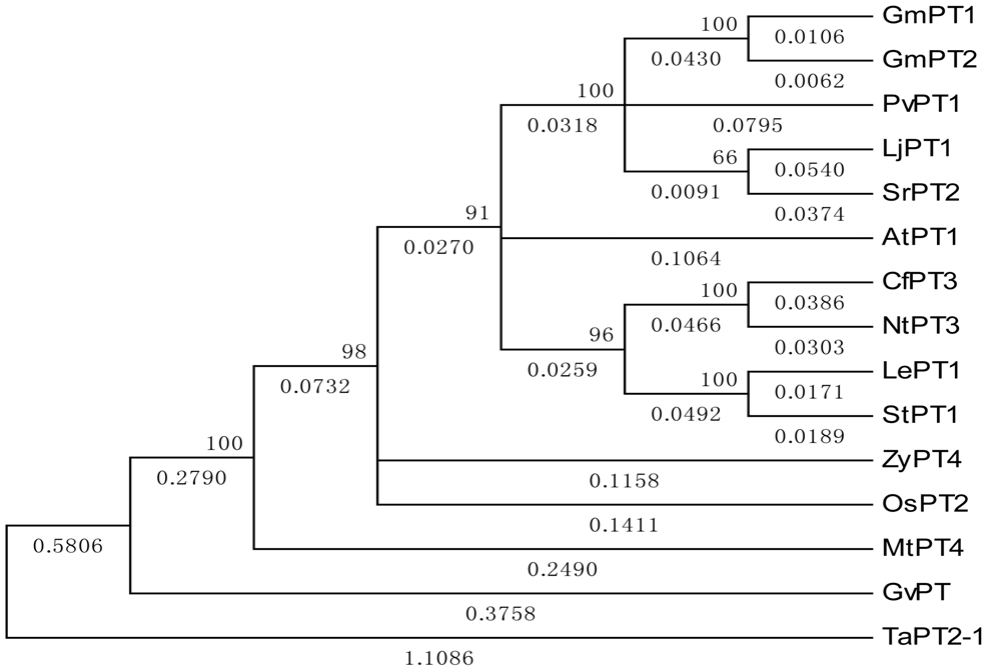
Phylogenetic relationship between GmPT1, GmPT2 and other plant and fungal Pi transporters. Proteins (and accession numbers): PHO84 (P25297) from *Saccharomyces cerevisiae*; GvPT (Q00908) from *Glomus versiforme*; GiPT (AAL37552) from *Glomus intraradices*; Pht1;1 (Y07682), Pht1;2 (Y07681) Pht1;3 (O48639) and Pht2;1 (CAC15560) from *Arabidopsis thaliana*; StPT1 (Q43650) and StPT2 (Q41479) from *Solanum tuberosum*; MtPT1 (O22301) and MtPT2 (O22302) from *Medicago truncatula*; LePT1 (O24029) and LePT2 (O22549) from *Lycopersicon esculentum*; LaPT1(AAK01938) and LaPT2 (AAK38197) from *Lupinus albus*; NtPT1(AAF74025) from *Nicotiana tabacum*; OsPT1(AAN39042) and OsPT2 (AAN39043) from *Oryza sativa*; and GmPT1 (HQ392508) and GmPT2 (HQ392509) from *Glycine max*.

Hydropathy plots of the deduced polypeptides suggest that GmPT1 and GmPT2 consist of 12 membrane-spanning regions (Figure 2), a feature shared by other Pi transporters, irrespective of the level of affinity [14], [15], [19], [20], [25], [26], [27], [28], [29], [30], [31], [32], [33], [34], [35].Computational modeling of the encoded proteins predicted a conserved secondary structure containing 12 transmembrane (TM)domains with a large hydrophilic loop between TM6 and TM7 (Figure 2A) and the hydrophilic N and C termini located in the cytoplasm (Figure 2B). The amino acid sequences are similar to those of the other members of the Pht1 family of Pi transporters (Figure 3). Several amino acid domains are highly conserved between these two Pi transporters and include sites for protein kinase C and casein kinase II-facilitated phosphorylation, as well as N-glycosylation (Figure 2B). The existence of a number of conserved putative phosphorylation sites present within the Pht1 family suggested that regulation of the transporters might occur at the post-translational level as well [6], [9].

### Subcellular localization of GmPT1 and GmPT2

**Figure 4:**
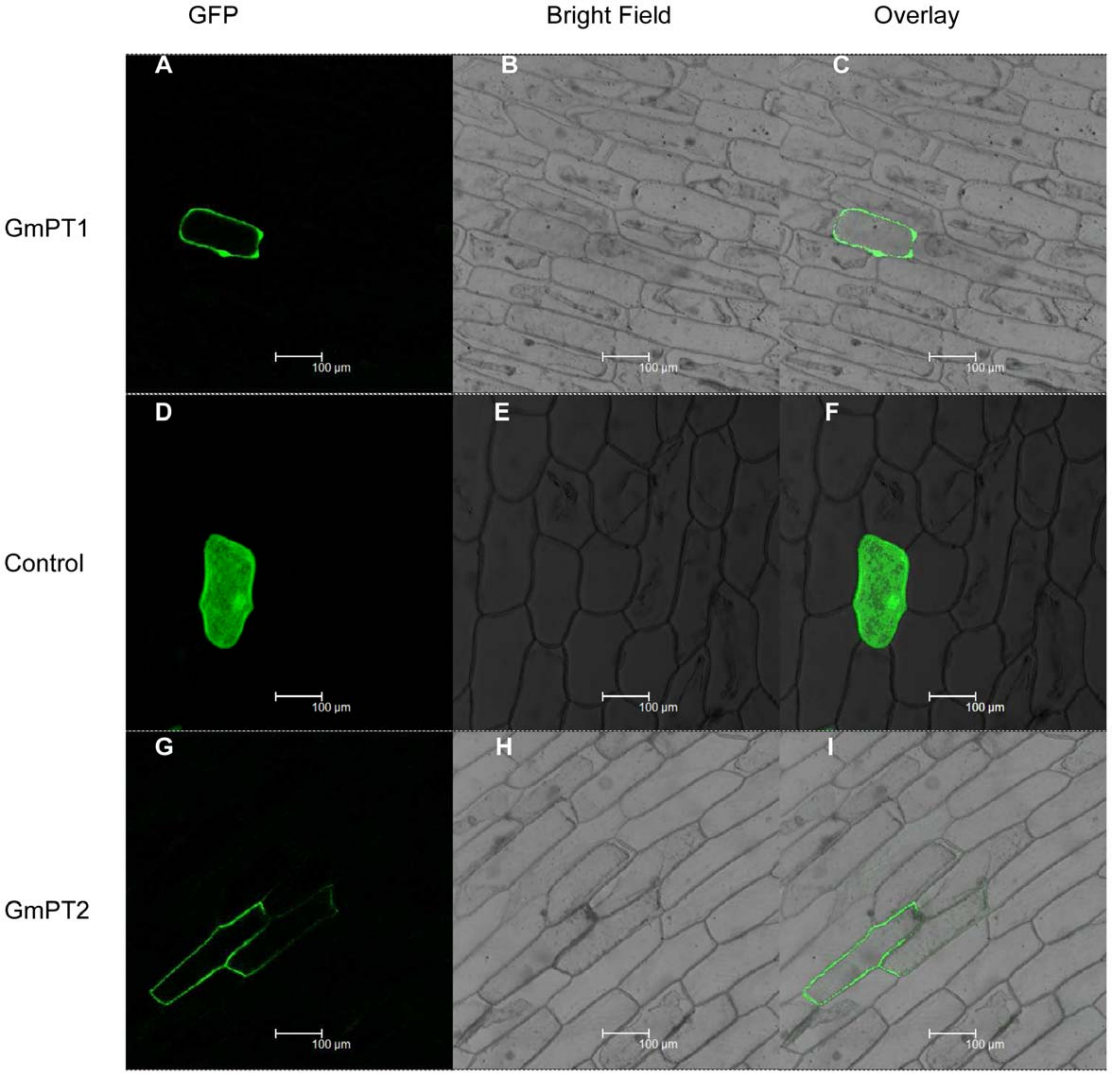
Subcellular localization of GmPT1/GFP and GmPT2/GFP fusion. Images showing onion epidermal cells expressing GmPT1/GFP (A–C), empty vector (D–F) and GmPT2/GFP (G–I) fusion protein examined under fluorescent-field illumination (A, D and G) to examine GFP fluorescence; under bright-field illumination (B, E and H) and by confocal microscopy for the overlay of bright and fluorescent illumination (C, F and I). The scale bars represent 100 µM.

The TBpred Prediction Server [36] (http://www.imtech.res.in/raghava/tbpred/) was used for searches that yielded unambiguous results with positive scores for the integral membrane protein (data not shown). To verify the subcellular locations of GmPT1 and GmPT2, a green fluorescent protein (GFP)-tagged gene was fused to the 3′ end of the open reading frame of the *GmPT1* or *GmPT2* genes. The chimeric genes were placed under the control of the CaMV35S promoter and the constructs were transformed into onion epidermal cell by particle bombarded. As a control, a second set of cells was bombarded with the empty vector pBI-121-GFP. The cells were then examined by confocal laser scanning microscopy to determine the location of the GmPT1/GFP and GmPT2/GFP fusion proteins. A clear GFP signal was observed at the periphery of cells bombarded with the GmPT1/GFP or GmPT2/GFP construction (Figure 4A–C and G–I for GmPT1 and GmPT2, respectively), whereas the signal was seen throughout cells expressing free GFP (Figure 4D–F). Localization of the GmPT1/GFP and GmPT2/GFP fusion proteins to the periphery of the cells indicated that the two proteins are targeted to the plasma membrane. This is consistent with the results of earlier biochemical studies and together these data suggest that the GmPT1 and GmPT2 proteins are located in the plasma membrane.

### Functional and Biochemical Analysis in Yeast

**Figure 5:**
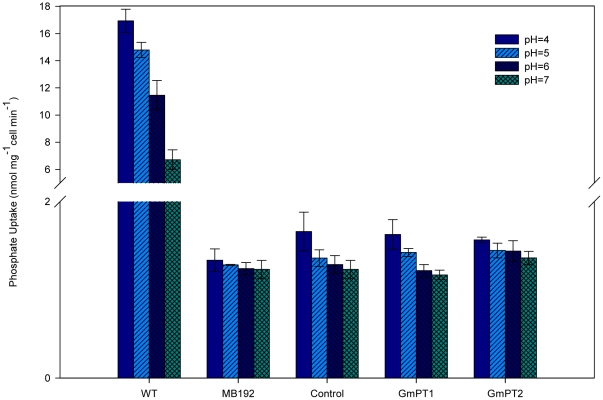
Inorganic phosphate (Pi) uptake as a function of external pH. Pi uptake rates for yeast MB192 cells expressing the indicated GmPT1, GmPT2 or carrying the control vector and wild type yeast cell were determined in medium at the indicated pH value. Values shown are the mean ± SE for three independent experiments.

We used uptake studies with inhibitors to confirm the pH dependence of Pi transport (Table 1). Pi transport activity was assessed at pH values in the range 4–7. Differences were detected in the activity profiles but the uptake rate was maximal at pH 4 and increased as the pH was reduced from 7 to 4 in each case (Figure 5). To investigate this influence of a proton motive force on Pi transport activity, the uncouplers 2,4-dinitrophenol (2,4-DNP) and carbonyl cyanide *m*-chlorophenylhydrazone (CCCP), which destroy pH gradients across membranes, were applied. DNP at a concentration of 100 µM reduced the Pi uptake rate to 79% (GmPT1) and 82% (GmPT2) compared with 100% uptake in the inhibitor-free control. The rate of uptake was reduced to 77% (GmPT1) and 80% (GmPT2) by 100 µM CCCP and, to 82% (GmPT1) and 83% (GmPT2) by 100 µM Vanadate, an inhibitor of P-type H^+^-ATPases. The transporter rate was decreased significantly compared to that in the control (Table 1). These results confirmed the hypothesis that Pi/H^+^ cotransport via GmPT1 and GmPT2 depends on the pH gradient across the cell membrane that is maintained by the endogenous plasma membrane H^+^-ATPases. Moreover, competition studies showed that different anions did not reduce the Pi uptake rate, demonstrating the high degree of specificity of GmPT1 and GmPT2 for Pi. Strains carrying the *GmPT1* or *GmPT2* cDNA generally uptake Pi at rates similar to those of the vector controls at millimolar concentrations of Pi (Figure 5).

**Table 1:** Pharmacology and specificity of GmPT1 and GmPT2.

Inhibitor	^32^P uptake (% of the control)	
	GmPT1Mean ± SE	GmPT2Mean ± SE
CCCP (10 µM)	93±0.04	96±0.06
CCCP (100 µM)	77±0.02	80±0.05
DNP (10 µM)	92±0.02	94±0.07
DNP (100 µM)	79±0.05	82±0.01
Vanadate (10 µM)	92±0.02	96±0.05
Vanadate (100 µM)	82±0.03	83±0.04
NH_4_Cl (5 mM)	78±0.08	76±0.05
KCl (5 mM)	75±0.03	72±0.07
NaAc (5 mM)	77±0.08	78±0.04

CCCP, carbonyl cyanide *m*-chlorophenylhydrazone.

DNP, 2,4-dinitrophenol.

Inhibitors were added to yeast cells 30 s before addition of labeled Pi. All assays were done at pH 4. Values for each treatment were derived from three independent measurements. Water was used as the control treatment.

**Figure 6:**
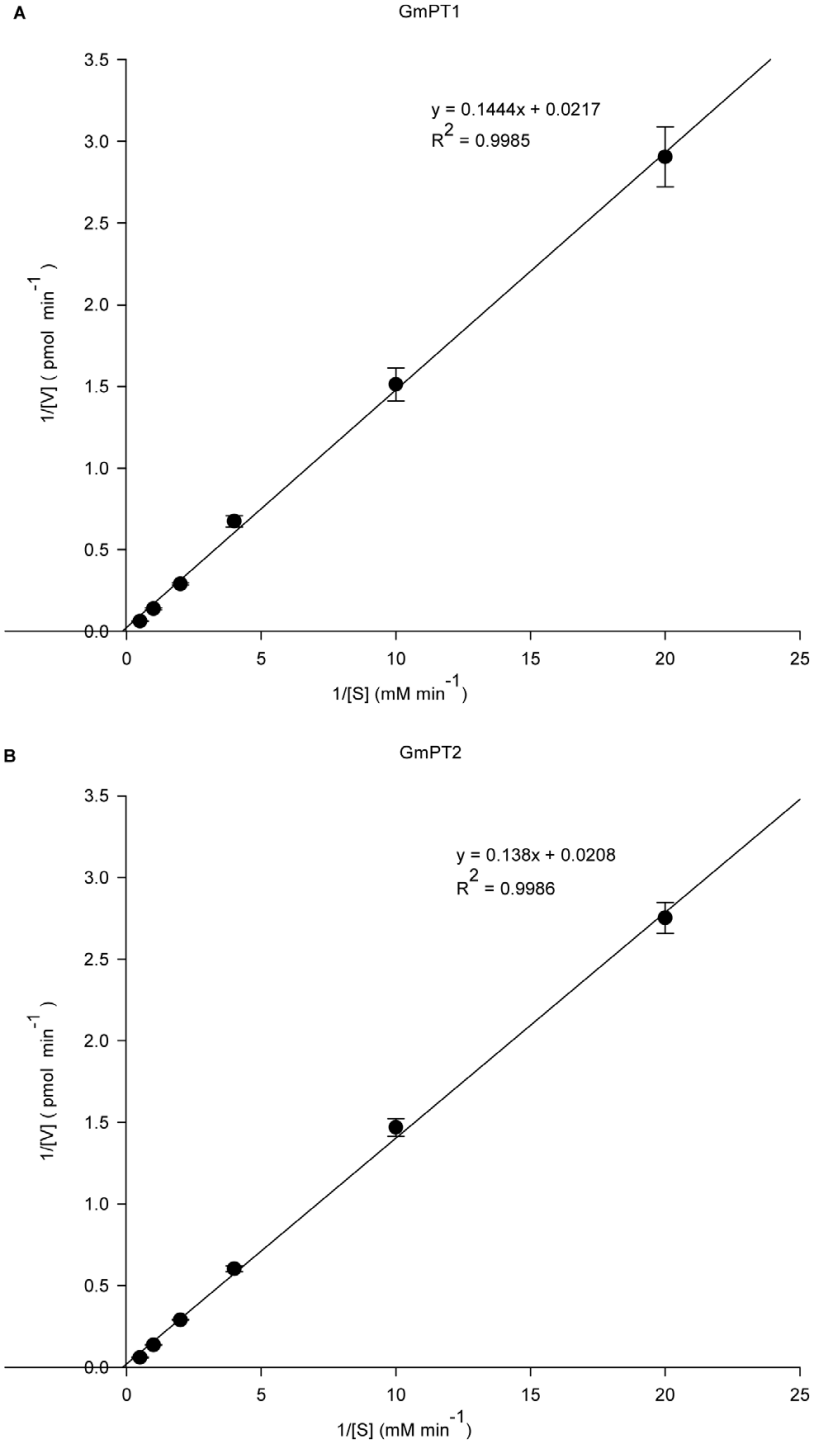
Lineweaver–Burk plots of GmPT1 and GmPT2. Lineweaver–Burk plot of Pi uptake of strains MB192-GmPT1 and MB192-GmPT2 versus external Pi concentrations that were used to estimate *K*_m_.

It was the pioneering work of Emmanuel Epstein that demonstrated ion uptake processes across the plasma membrane follow Michaelis–Menten kinetics [37], [38]. In uptake experiments with radioactive Pi, the rate of transport was linear with time during the first 5 min of uptake under the conditions applied [30], [31]. In three parallel experiments, the Lineweaver–Burk diagram, calculated using reciprocal uptake velocities at 5 min after addition of ^32^Pi, indicated that Pi uptake facilitated by GmPT1and GmPT2 followed Michaelis–Menten kinetics with an apparent *K*_m_ value of 6.65 mM and 6.63 mM, respectively, (Figure 6A and B). Thus, GmPT1 and GmPT2 are low-affinity Pi transporters that are dependent on the proton gradient across the plasma membrane.

### Expression pattern of *GmPT1* and *GmPT2*

**Figure 7:**
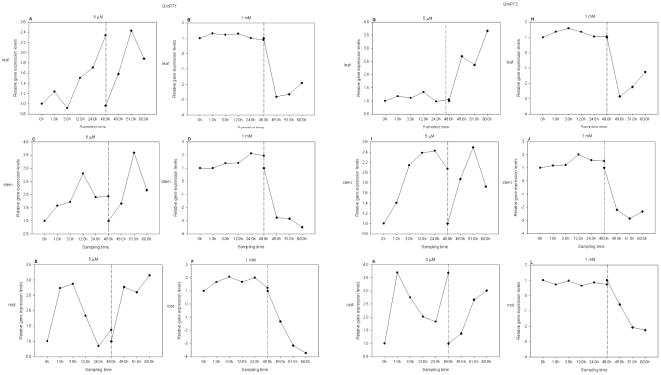
Expression levels of *GmPT1* (A–F) and *GmPT2* (G–L) during Pi treatment. The 7-day-old seedlings were grown by hydroponic culture with 0.5×Hoagland solution containing 5 µM Pi (A, C, E, G, I and K) or 1 mM Pi (B, D, F, H, J and L). Seedling tissues were harvested at 0, 1.0, 3.0, 12.0, 24.0 and 48.0 h after treatment (on the dash-dot line at the left). After treatment for 48 h, the deficient/sufficient Pi-treated seedlings were transferred to sufficient/deficient Pi in Hoagland solution, respectively. Seedling tissues were sampled at 0, 1.0 and 3.0 h after changing the nutrient solution (on the dash-dot line at the right). Leaf, A and B, G and H; stem, C and D, I and J; and root, E and F, K and L.

Root, stem and leaf tissues of 7-day-old soybean seedlings were used to examine the expression of *GmPT1* and *GmPT2* (Figure 1B). Expression of the two Pi transporters was enhanced in both root and shoot during the first 48 h of Pi starvation. The expression of *GmPT1* and *GmPT2* in seedling tissues was increased during the 3 h after the Pi-sufficient treated seedlings were transferred to a Pi-deficient solution at 48 h compared to the expression measured in Pi-sufficient plants (Figure 7A,C and E for *GmPT1* and G, I and K for *GmPT2*). The transcript levels of *GmPT1* and *GmPT2* were little changed in plants that were grown in half-strength nutrient solution for 7 days and then transferred to a Pi-sufficient solution. A decrease in the transcript abundance of *GmPT1* and *GmPT2* in the leaf, stem and root of hydroponically grown soybean seedlings was apparent within 3 h of Pi deprivation (Figure 7 B, D and F for *GmPT1* and H, J and L for *GmPT2*). In conclusion, the expression level of the two genes was not altered markedly and the change tendencies were complicated irrespective of how the seedlings were treated. Therefore, the *GmPT1* and *GmPT2* soybean Pi transporters were constitutively expressed.

## Discussion

### *GmPT1* and *GmPT2* are members of the Pht1 family

Our studies provide the first insights into the molecular nature of the proteins involved in phosphate transport in the soybean and reveal that soybean has phosphate transporters with sequence similarity to proton-coupled symporters from a large number of plants and fungi. These transporters belong to the phosphate: H^+^ symporter (PHS) transporter family of the major facilitator superfamily [39]. Phylogenetically, the Pi transporters in plants and fungi belong to a closely related family, even though the similarity between the plant transporters is significantly higher than that between plants and fungi transporters [14]. These genes have been grouped into the Pht1 family of proton–Pi cotransporters [40], which are energized by the plasma membrane proton ATPase [4]. In addition, at least one phosphorylation site and one N-glycosylation site among the potential protein modification sites are completely conserved in all plant transporters [14], [15], [26], [33].

### GmPT1 and GmPT2 are low-affinity Pi transporters

The results of uptake and kinetic studies led us to conclude that both GmPT1 and GmPT2 probably have a low affinity for Pi at millimolar concentrations, similar to the endogenous yeast low-affinity Pi uptake system. It has been reported that Δpho87Δpho89Δpho90Δpho91 cells in a wild type strain do not show any significant defect in Pi uptake under high-Pi conditions; meanwhile, the loss of at least one low-affinity Pi transporter could result in an insufficient Pi uptake similar to the case of PHO84 inactivation under Pi-limiting conditions [41]. These results revealed that the inactivation of low-affinity Pi transporters does not result in a substantial defect in Pi uptake, even though these proteins have been shown to play a role in Pi uptake.

The high-affinity Pi transporters are inducible in plants and fungi, whereas the low-affinity transporters are expressed constitutively. The Pht2 family in Arabidopsis is considered to be composed of low-affinity proton/Pi symporters, the expression of which is high in shoots and is not altered substantially during Pi starvation. The apparent low-affinity proton/Pi symporters that are highly expressed around vascular bundles suggests that those symporters play a role in loading shoot organs with Pi [40].

### The profile of a Pi transporter

**Figure 8:**
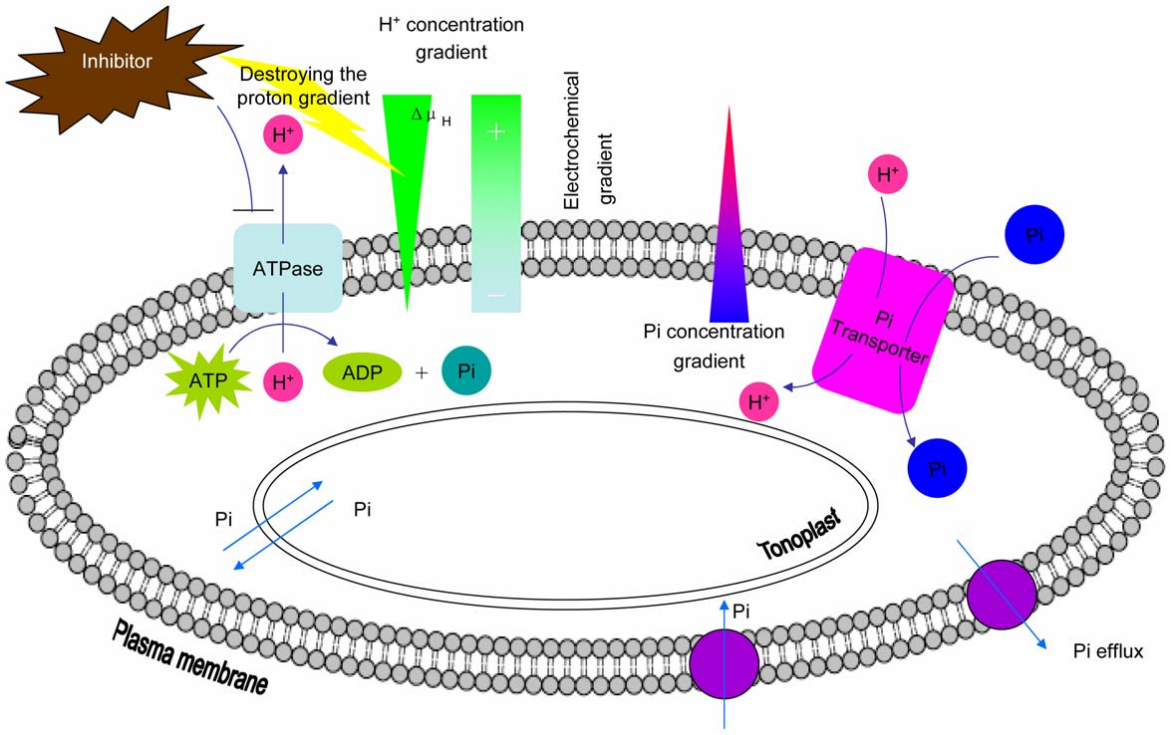
The Pi transporter mechanism in the plant cell. A membrane-integral proton ATPase undirectionally extrudes protons (H^+^) at the expense of ATP. The proton concentration gradient and membrane potential generated constitute a proton electrochemical potential (Δμ_H_) across the membrane. Proton movement along the concentration and electrical gradients facilitates Pi movement by Pi transporters against a steep concentration gradient. Meanwhile, the efflux mechanism helps to maintain Pi homeostasis in the cells.

The uptake and distribution of Pi in plants requires multiple Pi transport systems that must function in concert to maintain homeostasis throughout growth and development. Phosphate uptake in plants is an energy-mediated co-transport process driven by a proton gradient generated by plasma membrane H^+^-ATPases [6], [42], [43], [44]. At millimolar concentrations of intracellular Pi, Pi uptake is accomplished by transport of the anion across the membrane coupled to the transport of protons (H^+^-symport). Therefore, the driving force for Pi influx is the proton gradient generated by the H^+^-ATPases (Figure 8). It has been assumed that plant Pi transporters are proton/Pi co-transporters with a stoichiometry of 2–4 H^+^/Pi [2]. By complementation of a knock-out of endogenous high-affinity Pi transporters of various yeast mutants, or by measuring the increase of Pi uptake in transformed plant cells, several Pi transporters of many plant species have shown common properties, indicating that there is an electrochemical proton gradient across the plasma membrane [6], [20], [26], [27], [30], [31], [45].

The transport of Pi across plant membranes driven by the proton/Pi co-transporter mechanism is pH dependent. The observed increase of Pi uptake rates in response to decreasing pH is consistent with the operation of a proton/Pi symporter. Our experiments show that the peak of Pi uptake is at pH 4.0 (Figure 5), which reflects the fact that the transport mechanism is a proton/Pi symport. The reduced uptake rate (Table 1) in the presence of uncouplers of pH gradients across membranes, such as DNP and CCCP, favors the latter interpretation. This view is supported by the finding that addition of glucose before the uptake experiment with radioactive Pi enhances the uptake capacity of transformants. This effect could be caused by an enhanced proton extrusion that might result from preincubation with glucose. We have demonstrated the Pi transport activity of GmPT1 and GmPT2, which are low-affinity transporters in soybean, are dependent on the electrochemical gradient of protons as indicated by the pH dependence and the pharmacological assay. Transiently expressed GFP protein fusions provide direct evidence that the two Pi transporters are located in the plasma membrane (Figure 4). The results suggested also that the encoded proteins function in the plasma membrane.

### Low-affinity Pi transporters play important roles in Pi homeostasis within plants

The transport of Pi across membranes is a pivotal step in the regulation of Pi use. Plants require multiple Pi transport systems to facilitate acquisition of Pi from diverse environments and to enable its subsequent transport to all of the cells and subcellular compartments of the plant [8]. The low concentration of Pi commonly found in the soil solution [8] has led to the hypothesis that only high-affinity Pi transporters can function for the uptake of Pi across the plasma membrane of root epidermal cells, whereas the low-affinity Pi transporters could be responsible for transport of Pi within the plant [20]. Initially, Pi is transported into root epidermal cells and subsequently loaded into the xylem for translocation to the aerial portions of the plant. Under conditions of Pi deficiency, Pi can be retranslocated from shoot tissues to the roots via the phloem [2], [4], [11].

Two *A. thaliana* mutants exhibiting altered phosphate accumulation have been described, among which the *pho1* mutant is deficient in the translocation of Pi from the roots to the shoots [46], whereas a mutation at the *pho2* locus resulted in excessive accumulation of Pi in the leaves [47], [48]. OsPT2 is a low-affinity Pi transporter that is expressed in the root stele and leaf phloem and xylem. On the basis of its tissue-specific expression pattern, OsPT2 is assumed to function in translocation of stored Pi in rice [21]. Over-expression of *OsPT2* (*PT2(O)*) in transgenic plants resulted in accumulation of excess shoot Pi and growth retardation similar to that of rice *pho2* mutants under Pi-sufficient conditions. There is no significant difference in the concentration of Pi in either shoot or root between wild type and *PT2(O)* under Pi-deficient conditions. These results suggest that over-expression of *OsPT2* increases Pi uptake and translocation of Pi from root to shoot, resulting in the accumulation of excess Pi in shoots under abundant Pi conditions [49]. Knock-down of *OsPT2* transgenic line *r2-1* have shown that the concentration of Pi in the shoot is much lower than that of the wild type [21]; therefore, OsPT2 is responsible for translocation of the stored Pi in the plant.

When the supply of Pi is limited, plants grow more roots, increase the rate of Pi uptake by roots from soil solution, retranslocate Pi from older leaves, and deplete the vacuolar stores of Pi. There is also significant retranslocation of Pi in the phloem from older leaves to the growing shoot and from the shoot to the root. In Pi-deficient plants, the restricted supply of Pi to the shoots from the roots via the xylem is supplemented by increased mobilization of stored P in the older leaves and retranslocation to both the younger leaves and growing roots. This process involves both the depletion of Pi stores and the breakdown of organic P in the older leaves. A curious feature of Pi-starved plants is that approximately one-half of the Pi translocated from the shoot to the root in the phloem is then transferred to the xylem and recycled back to the shoot [4]. Low-affinity Pi transporters in the Pht1 family are now thought to play this role in translocation of Pi within the plant, and this has been inferred from the spatial expression of these genes in several different plant species [7], [30], [49], [50]. In conclusion, low-affinity Pi transporters play important roles in Pi homeostasis within plants.

### Prospects

Under conditions of Pi starvation, soybean can display its unique strategies to improve its acquisition and remobilization of Pi. In addition, the physiological and molecular processes in soybean under conditions of Pi deficiency appear more complex. Therefore, a global survey of Pi transporter expression in response to Pi starvation is necessary to understand the network of gene expression related to Pi acquisition, translocation, recycling and signal transduction. In this study, we analyzed the temporal and spatial expression patterns of Pi transporters from soybean seedlings subjected to Pi starvation.

A BLAST search of the soybean genome, combined with cDNA cloning, showed that soybean possibly contains nine Pi transporter genes. In this study, we analyzed the expression levels of *GmPT1* and *GmPT2*, but there could be other Pi transporters in soybean, which raises the question of whether other Pi transporters can affect Pi acquisition, translocation and remobilization and what is the relative contribution of these genes to overall Pi transporter function in plants? Multiple Pi transporter genes could result in finer control over protein expression; if so, how does each of these genes respond to deficiency of Pi stress? Future studies of the expression of all soybean Pi transporters in response to different concentrations of Pi could address these questions, providing better understanding of the function of Pi transporter genes in soybean.

## Materials and Methods

### Plant material and growth conditions

Surface-sterilized soybean seeds (*G. max* cv. gantai) were sown in sterile, acid-washed quartz sand irrigated with 0.5×Hoagland solution containing 5 µM Pi. The seedings were maintained in a growth chamber with 70% relative humidity and a cycle of 16 h light at 29°C/8 h dark at 23°C. After 7 days, fresh roots were harvested for gene cloning. At the same time, whole plants were transferred to 0.5×Hoagland solution for a Pi-deficiency time-course experiment.

For the experiment, 7-day-old seedlings were grown by hydroponic culture with 0.5×Hoagland solution containing 5 µM Pi (Pi deficient) or 1 mM Pi (Pi sufficient), respectively. All seedling tissues were harvested at 0, 1.0, 3.0, 12.0, 24.0 and 48.0 h after treatment. After Pi deficient/sufficient treatment for 48 h, the seedlings were transferred to Hoagland solution with sufficient/deficient Pi, respectively. Seedling tissues were sampled at 0, 1.0, and 3.0 h after transplanting: the time points for sampling were 0, 1.0, 3.0, 12.0, 24.0, 48.0, 49.0, 51.0 and 60.0 h.

### Gene Cloning

Using the *OsPT2* nucleotide sequence (accession number AF536962) as the query, a BALSTN [24] search was done on the web page of the phytozome (http://www.phytozome.net/search.php?show=blast) to identify sequences containing *OsPT2* orthologs in the soybean genomic database. This resulted in the identification of two cDNA clones designated *GmPT1* and *GmPT2*. Two pairs of primers were used for PCR amplification: for *GmPT1*

forward 5′-CAGGTAGCTGAGTTAGTGAGTGA-3′

reverse 5′-CACGTATGATTTAGACAACACTTC-3′

for *GmPT2*

forward 5′-CAGGTAGCAGAGTTAGTGAGTAAT-3′

reverse 5′-ACAAGAATGAAATACACACCC-3′

Full-length cDNA was amplified from the root cDNA template, using the primers at the end of the cDNA sequence, and then cloned into the pMD-19 Simple T vector (Takara) for sequence verification.

### Sequence Analysis

Sequence analysis was done with ANTHEPROT [51], Lasergene version 7.0.1 and DNAMAN version 6.0.3.93 software. Transmembrane regions and subcellular localization were predicted by HMMTOP [52] (http://www.enzim.hu/hmmtop/index.html) and the TBpred prediction server [36] (http://www.imtech.res.in/raghava/tbpred/), respectively. We used ScanProsite to scan the protein sequences for the occurrence of patterns stored in the PROSITE database [53]. The ScanProsite tools are available on the ExPaSy Molecular Biology of Geneva (Switzerland) website (http://expasy.org/tools/scanprosite/).

Multiple sequence alignment was done with ClustalW [54]. MEGA 4 [55] was used for analysis of the phylogenetic relationships of GmPT1 and GmPT2 and other Pi transporters. The evolutionary history was inferred using the neighbor-joining method. The bootstrap consensus tree inferred from 1000 replicates was taken to represent the evolutionary history of the taxa analyzed. Branches corresponding to partitions reproduced in less than <60% bootstrap replicates were collapsed. The percentage of replicate trees in which the associated taxa clustered together in the bootstrap test (1000 replicates) are shown next to the branches (next to the branches). The evolutionary distances were computed using the Poisson correction method and are in units of the number of amino acid substitutions per site. All positions containing gaps and missing data were eliminated from the dataset (complete deletion option). There are 460 positions in the final dataset.

### Subcellular Localization

GFP was fused to the 3′ ends of *GmPT1* and *GmPT2*. When expressed in onion epidermal cells, these gene fusions gave rise to Pi transporter::GFP fusion proteins. A PCR-generated *Xba* I–*Bam*H I fragment containing the open reading frame of *GmPT1* and the *Xba* I–*Xba* I fragment containing the open reading frame of *GmPT2* were subcloned in-frame upstream of the GFP gene in plasmid pBI-121-GFP. The primers were:

for *GmPT1*

forward 5′- GCTCTAGAATGGCGGGAGGACAACTAG -3′

reverse 5′- CGGGATCCAACTGGAACCGTCCTA-3′

for *GmPT2*

forward 5′-GCT CTAGAATGGCAGGAGGACAACTAG-3′

reverse 5′-GCT CTAGAAACTGGAACCGTCCTAGC-3′

Expression of the gene fusions was controlled by the CaMV35S-promoter.

DNA of the chimeric genes CaMV35S-*GmPT1* and CaMV35S-*GmPT2* and the pBI-121-GFP empty vector were introduced into onion epidermal cells by a particle bombardment system (Biolistic PDS-1000/He System; BioRad, USA) according to the manufacturer's instructions. Bombarded samples were kept in the dark at room temperature for ∼24 h and then examined under a Leica TCS SP2 confocal microscope.

### Yeast Manipulations and Pi Uptake Assays

The open reading frames of *GmPT1* and *GmPT2* were separately subcloned into yeast expression vector p112A1NE [56] to create *GmPT1/*p112A1NE and *GmPT2/*p112A1NE, where expression of *GmPT1* or *GmPT2* gene was driven by the alcohol dehydrogenase promoter 1 (ADH1). These constructs were transformed into the yeast mutant MB192 (***MATa*** *pho3-1* Δ*pho84::HIS3 ade2 leu2-3*,*112 his3-532 trp1-289 ura3-1*, *2 can1*) [28] as described [57].

The yeast cells were grown until the logarithmic phase (when the absorbance at 600 nm was 1.0) on YNB liquid medium (Difco, Chemie Brunschwig AG, Basel, Switzerland), harvested, washed three times with Pi-free medium (YNB medium containing an equimolar concentration of potassium chloride instead of potassium phosphate), then suspended in the same medium and incubated at 30°C for 10 min. Different extracellular pH values in the range 4.0–7.0 were used for the pH-dependent Pi uptake experiments. Washed and Pi-starved cells were suspended and activated with 20% (w/v) glucose to guarantee optimal energization of the plasma membrane to 5%. Then 1 ml of 1 mM ^32^Pi (final concentration of Pi 0.25 mM) was added, mixed and the cells were incubated with shaking at 30°C for 5 min. Uptake was stopped by addition of 4 ml of ice-cold water and the cells were harvested immediately on glass microfiber filters (Whatman® GF/F grade) by vacuum filtration. The filters were washed twice with 4 ml of ice-cold water then transferred to scintillation vials and radioactivity was measured by a Beckman LS 6500 Scintillation Counter. Six different concentrations of Pi (2000, 1000, 500, 250, 100 and 50 µM) were used to derive the value of *K*_m_ from the double reciprocal Lineweaver–Burk plot, which is less susceptible to skewing as a consequence of the multiple kinetics components; therefore, *K*_m_ is an aggregate value reflecting the contribution of many individual kinetic constants [58]. For inhibition studies, the reagents given in Table 1 were added 30 s before addition of the labeled Pi. Mes(2-(*N*-morpholino)ethanesulfonic acid) buffer at a final concentration of 25 mM was used to determine transport activity at different pH values.

### Quantitative real-time RT-PCR (qRT-PCR)

RNA was extracted from root and shoot samples using TRIzol® reagent (Invitrogen, Carlsbad, CA, USA). RT-PCR for the target genes, *GmPT1* and *GmPT2* and *cons7* (accession number AW310136) [59] using gene-specific primers followed the protocol as described [60]. PCR was done in triplicate using a reaction solution containing TaqMan buffer, 0.4 µM forward and reverse primers and 0.3 µM probe was done with the ABI 7500 Fast Real-Time PCR system. (Applied Biosystems). Expression levels were normalized according to *cons7* and fold change was calculated using the 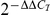 method [61]. The following gene-specific primers and probe were used for real-time RT-PCR:

for *GmPT1*

5′-CTTATGCTTATGGTTCTGTGTTCC-3′,

5′-CAGACATAATTGTAGCTGATAGAGG-3′

5′-(FAM) CACCACCAATCCCAAAGTCAAGCCA (TAMRA)-3′

for *GmPT2*

5′-GGCTTAACTCTTATGCTTATGGTTG-3′,

5′-CATGATTGTAGCTGATAGAGGGTAG-3′

5′-(FAM) CACCACCAATCCCAAAGCCAAGCCA(TAMRA)-3′

for *cons7*

5′- TATAAACCTGGAGGATGCACTAGC-3′

5′- GTACATGGGAACCGTCATTCATC-3′

5′-(FAM) AACGGAAGCCTCAGAACCACACTTG(TAMRA)-3′
